# Handling Ibuprofen Increases Pain Tolerance and Decreases Perceived Pain Intensity in a Cold Pressor Test

**DOI:** 10.1371/journal.pone.0056175

**Published:** 2013-03-04

**Authors:** Abraham M. Rutchick, Michael L. Slepian

**Affiliations:** 1 Department of Psychology, California State University, Northridge, Northridge, California, United States of America; 2 Department of Psychology, Tufts University, Medford, Massachusetts, United States of America; Université catholique de Louvain, Belgium

## Abstract

Pain contributes to health care costs, missed work and school, and lower quality of life. Extant research on psychological interventions for pain has focused primarily on developing skills that individuals can apply to manage their pain. Rather than examining internal factors that influence pain tolerance (e.g., pain management skills), the current work examines factors external to an individual that can increase pain tolerance. Specifically, the current study examined the nonconscious influence of exposure to meaningful objects on the perception of pain. Participants (N* = *54) completed a cold pressor test, examined either ibuprofen or a control object, then completed another cold pressor test. In the second test, participants who previously examined ibuprofen reported experiencing less intense pain and tolerated immersion longer (relative to baseline) than those who examined the control object. Theoretical and applied implications of these findings are discussed.

## Introduction

It was recently estimated that 100 million Americans experience chronic pain [1]. Chronic pain contributes to rising health care costs and lost productivity estimated at $560–$635 billion annually, as well as a significant impact on quality of life [1]. Thus, pain is an important health outcome that has inspired an extensive body of research. This work has elucidated the determinants of pain tolerance, described pharmacological and surgical treatments for pain, and described ways that psychological interventions can enhance pain management [2]. Beyond demonstrations of the positive influences of social support [3], extant research on psychological interventions for pain has focused primarily on developing a repertoire of skills that individuals can apply to manage their pain (e.g., enhancing emotion regulation and coping skills, relaxation, gaining insight into pain [4]–[7]). Much has been revealed, then, about what might be termed “internal” influences on pain, such as personally implemented techniques and individual differences in pain tolerance [8]. In contrast, the current work examines factors external to an individual that can increase pain tolerance: specifically, we examine how pain tolerance can be promoted by external environmental cues.

It is well-established that exposure to meaningful objects can change behavior in accordance with learned associations with those objects. For example, seeing money makes people act more self-sufficiently [9], the presence of guns increases aggressive responses to provocation [10], and using red pens induces evaluators to find more errors and grade more harshly [11]. In fact, there is evidence to suggest that seeing pain medication can lead to reduced pain. Previous research [12] demonstrated that exposure to pain medication, relative to a control object, led to lower ratings of pain intensity after 30 seconds of a cold pressor task. The current work sought to replicate and extend that research with several methodological refinements. First, the current study utilized both pre- and post-measures of pain intensity, allowing for the examination of changes in pain tolerance and intensity following exposure to an over-the-counter analgesic. Second, the current study utilized time-sensitive and nonverbal reports of pain, allowing for a more dynamic and real-time measure of pain. Third, adapting paradigms from the social psychology and priming literature, we developed a more naturalistic paradigm for exposing participants to the analgesic. Last, expectations regarding stimulus exposure were measured. Participants were randomly assigned to examine either an over-the-counter analgesic (branded ibuprofen) or a control object, and their pain tolerance and perceptions of pain intensity were assessed using a cold pressor test. It was hypothesized that participants who examined ibuprofen would tolerate the cold pressor for longer and would report experiencing less intense pain.

## Methods

### Ethics Statement

Participants provided written informed consent. These procedures were approved by the Institutional Review Board at California State University, Northridge.

### Participants and Procedure

Sixty undergraduates participated in the study to partially fulfill a course requirement. They were screened for recent use of painkillers and the presence of medical conditions that contraindicate participation in a cold pressor task (hand or wrist pain, cardiovascular problems, arthritis, diabetes, chronic pain, fibromyalgia, Reynaud’s disease). Six participants were excluded on the basis of this screening, yielding a final sample of 54 participants (91% female, *M*_age_ = 19.0). They were told that the experiment examined relations between various senses, and anticipated completing tests of haptic, visual, and auditory sensitivity.

Because pilot testing revealed high individual differences in cold pressor tolerance in this sampling frame, a pretest-posttest design [13] was used. Participants first completed a baseline cold pressor test, in which they immersed their left hands in a room temperature bath for two minutes, then in a cold (0–2°Celsius) circulating bath for as long as possible. Each participant ended the cold pressor task by removing his or her hand from the bath when the pain became too uncomfortable. Simultaneously, participants used their right hands to indicate how much pain they felt by pointing to a modified version of the Faces Pain Scale [14]. This scale contains six faces whose expressions show varying degrees of pain; it ranges from 0 (no pain) to 10 (as much pain as you can imagine). Even numbers correspond to the faces; odd numbers represent the spaces between the faces. Without alerting participants, the experimenter recorded pain intensity every ten seconds while participants pointed toward the pain scale. This yielded an online measure of pain intensity [15] rather than the retrospective judgment that is often obtained [16], [17]. Pain tolerance, measured by immersion time, was also recorded. For safety, participants who still had their hands immersed after three minutes were asked to stop.

After the cold pressor test, participants rested for two minutes. They were told that during this recovery period they would complete another study (ostensibly for another researcher) which entailed evaluating the design of several products. This cover story was supported by the fact that the laboratory next door (in the same suite) was a human factors laboratory that frequently conducted studies of this kind. Participants responded to a five-item questionnaire (e.g., “How well-designed is this product’s container?”; see Appendix S1) about each of three objects: a water bottle, a stapler in its box, and a container of either branded ibuprofen or microwaveable noodles (a control object, also consumable); the third object was randomly assigned to participants. This assignment yielded 29 participants in the control condition (25 female, 4 male; *M_age_* = 18.86) and 25 participants in the ibuprofen condition (24 female, 1 male; *M_age_* = 19.24). Next, participants completed the PANAS affect measure [18]. Last, participants completed a second cold pressor test, identical to the first. Participants were then probed for suspicion (during which no participants associated the product evaluation task with the cold pressor test), debriefed, and dismissed.

The experimenters who administered these procedures were aware of which objects participants were handling (thus, they were not blind to condition). However, they were unaware of the specific hypotheses under investigation (that is, they were blind with respect to the directionality of the predicted effect). Moreover, experimenter-participant interactions were governed by a specific script to minimize potential experimenter expectation effects (see also Appendix S2 for a discussion of these expectations).

## Results

Because significant skew was indicated by Shapiro-Wilk tests (*W = *.70, *p<*.01 and *W = *.74, *p<*.01, respectively, for tolerance in the baseline and second cold pressor tests) pain tolerance times were log-transformed to correct for skew before conducting tests of statistical significance. Transformation corrected this skew (*W = *.98, *p = *.44 and *W = *.97, *p = *.19, respectively); analyses of untransformed pain tolerance yielded a comparable pattern of results.

A mixed-model analysis of variance revealed no main effect of Exposure (ibuprofen vs. noodles), *F*(1,52) = .26, *p = *.61, *η^2^_p_*<.01, or Trial (baseline CPT vs. second CPT), *F*(1,52) = .01, *p = *.91, *η^2^_p_*<.01. As predicted, there was a significant Exposure × Trial interaction, *F*(1,52) = 5.96, *p = *.02, *η^2^_p_* = .10; relative to baseline tolerance, participants exposed to ibuprofen tolerated immersion longer in the second cold pressor test than participants exposed to noodles, confirming the primary hypothesis. All means and standard deviations are reported in Table 1.

**Table 1:** Tolerance, intensity and affect means and standard deviations.

		Ibuprofen		Noodles	
		Baseline	Second CPT	Baseline	Second CPT
Tolerance (s)	*M* (*SD*)	40.12 (46.57)	45.28 (49.64)	42.03 (35.04)	41.83 (38.31)
Intensity (FACES)	10 sec (n = 54)	4.56 (3.08)	3.84 (3.36)	4.00 (2.45)	4.62 (3.21)
	20 sec (n = 41)	5.67 (3.09)	4.50 (3.67)	5.74 (2.72)	6.26 (2.78)
	30 sec (n = 27)	6.18 (2.09)	4.18 (2.60)	6.50 (2.58)	6.25 (2.72)
	40 sec (n = 16)	7.00 (1.85)	5.25 (2.12)	5.25 (1.83)	5.50 (1.41)
Positive Affect (PANAS)		28.16 (8.09)		30.72 (5.52)	
Negative Affect (PANAS)		14.92 (3.86)		13.59 (3.02)	

Parallel analyses were conducted on ratings of pain intensity. At 10 seconds post-immersion, there was no main effect of Exposure, *F*(1,52) = .02, *p = *.89, *η^2^_p_*<.01, or Trial, *F*(1,52) = .03, *p = *.85, *η^2^_p_*<.01. As predicted, there was a significant Exposure × Trial interaction, *F*(1,52) = 6.28, *p = *.02, *η^2^_p_* = .11; relative to baseline, participants who examined ibuprofen reported experiencing less intense pain in the second cold pressor test than those who examined noodles. Parallel effects were observed at 20 seconds post-submersion (no main effect of Exposure, *F*(1,39) = 1.05, *p = *.31, *η^2^_p_* = .03, or Trial, *F*(1,39) = 0.86, *p = *.36, *η^2^_p_* = .02, but the predicted Exposure × Trial interaction, *F*(1,39) = 5.90, *p = *.02, *η^2^_p_* = .13). At 30 seconds post-submersion, there was no main effect of Exposure, *F*(1,25) = 1.77, *p = *.20, *η^2^_p_* = .07, but a main effect of Trial emerged, *F*(1,25) = 6.93, *p = *.01, *η^2^_p_* = .22; participants reported less intense pain in the second CPT. Importantly, the predicted Exposure × Trial interaction remained, though its statistical significance became marginal, *F*(1,25) = 4.19, *p = *.05, *η^2^_p_* = .14). At 40 seconds post-submersion, there was again no main effect of Exposure, *F*(1,14) = 0.81, *p = *.38, *η^2^_p_* = .06, and the main effect of Trial remained, *F*(1,14) = 4.07, *p = *.06, *η^2^_p_* = .23; again, the predicted Exposure × Trial interaction was significant, *F*(1,14) = 7.23, *p = *.02, *η^2^_p_* = .34). After 40 seconds, too few participants remained to conduct reliable inferential tests. Finally, object exposure did not significantly influence reports of positive affect, *t*(52) = 1.38, *p = *.18, nor negative affect, *t*(52) = 1.42, *p = *.17. This suggests that the observed effects were not driven by changes in affect.

An alternative way to examine the impact of handling ibuprofen is to compare, across conditions, the number of participants who experienced meaningfully less pain in the second cold pressor test. This approach is analogous to clinical and epidemiological assessments of treatment efficacy that compare, between groups, the number of patients who show clinically significant improvement. We conducted such a comparison as a supplementary analysis. A composite score of change in intensity across all time points was computed, yielding an average change in intensity per time point for each participant. A reduction of pain intensity of at least 1.3 scale points per time unit was considered a meaningful improvement. This cutoff was chosen because, in previous research using a similar pain scale [19], it represented a small unit of meaningful improvement as indicated by chronic pain sufferers as they experienced pharmacological analgesics. Examining these scores revealed that only 3 of the 29 (10.3%) participants who examined noodles felt less pain during the second cold pressor test than the first, whereas 10 of the 25 (40.0%) participants who examined ibuprofen felt less pain in the second test than the first. This difference was statistically significant (*OR* = 3.87, *χ^2^ = *6.46, *p = *.01).

## Discussion

Handling a bottle of ibuprofen increased pain tolerance and decreased perceived pain intensity in a cold pressor test. As this influence was received nonconsciously (i.e., participants did not expect this procedure to confer pain tolerance), this finding offers support for the proposal that nonconscious priming interventions could have clinically relevant consequences. Although the intervention examined in the present study is not directly applicable to clinical practice, it underscores the powerful impact of objects and other innocuous-seeming contextual influences on cognition and behavior, suggesting that nonconscious interventions could indeed be considered for incorporation into health care services.

Prior work examining the impact of objects and stimuli in the environment has demonstrated that a diverse set of behaviors and cognitions are influenced by the meanings associated with such objects. Aggression, self-sufficiency, evaluation of others’ work, competitiveness, goal pursuit and creativity can all be swayed by objects in the environment [9]–[11], [20]–[24]. Additionally, framing can influence how painful stimuli are rated (e.g., work on placebo analgesia [25], [26], and framing and stimulus exposure can influence pain sensitivity [27]–[30]. There is evidence that exposure to pain medication can reduce ratings of pain intensity [12]. These studies found that exposure to over-the-counter analgesics led to reduced ratings of pain intensity at 30 seconds post-immersion, but there was no effect of exposure was found for pain tolerance. The current research follows that line of work by implementing several methodological refinements. First, it attempted to reduce demand characteristics by using a more naturalistic procedure, adapted from social psychological paradigms, to expose participants to the primes, along with a more elaborate cover story. Second, it utilized more sensitive time measures, a pre- and post- measure of pain, and nonverbal reports of pain, with the goal of reducing experimenter effects and providing more information about the time course of the effect. Third, it directly examined participant expectations about stimulus exposure, and showed that the observed analgesic effect was not consistent with these expectations. Together with the seminal work on this theme, the current studies demonstrate that objects in the environment can nonconsciously decrease pain sensitivity and increase pain tolerance.

A recent call has been made [31] for the development of more efficient interventions that address the vast need for mental health care. Priming-based interventions represent one way to approach this issue [32]; such interventions can feasibly be administered via websites or smartphones at a wide scale. That environmental stimuli, such as the ibuprofen exposure effect documented here, can influence subjective experiences of pain holds promise for realizing clinical applications of priming (see [33], [34]). The current findings, then, provide evidence suggesting that priming interventions could conceivably be applied to improve clinical outcomes. Future research is needed to examine whether procedures such as these can be used in clinical settings and on clinical samples, as the precise one used in the current work is not likely to be directly applicable to clinical practice. Other priming interventions, such as subliminal lexical or image priming, could perhaps be delivered via computer or smartphone applications. Given the subtlety required by this approach, such interventions would need to be designed with creativity and care. For example, they could conceivably be delivered in the context of another application, or recipients could be given a cover story that masks the specific nature of the intervention.

In addition to potential clinical implications, the current work has theoretical implications for work within the priming tradition, as well as the role of expectancies in the placebo effect. First, a variety of behaviors and cognitions have been demonstrated to be influenced by priming (see [35], [36]); the current finding shows that subjective experiences of physical sensations can also be nonconsciously swayed by stimuli in the environment. Second, regarding the placebo effect, although placebo effects can occur both without conditioning (e.g., novel drugs having different effects depending on the instructions that accompany them [37]) and without expectancies (e.g., placebo effects observed in rats [38]), evidence for expectancy-free placebo effects in humans is limited. Extant research demonstrating such effects has generally included explicit provision of treatments even in the control condition (i.e., placebo treatments). For example, an innovative series of studies has shown that patients conditioned with a drug that (imperceptibly to them) depressed respiratory function also showed depressed respiratory function in response to a placebo [39], [40], and that this effect is insensitive to changes in instructions [41]. Although patients’ expectancies were not specific to respiratory function, their knowledge that they had received a drug implies an expectation that they would experience a physiological effect – and that expectancy, associated with the respiratory response by classical conditioning, could have played a role in driving the observed changes. As participants in the current study did not believe that the ostensible product-design task could confer analgesic benefits, there were no expectations of treatment efficacy to account for the observed effect. Indeed, an additional study showed that participants believed handling ibuprofen to be unlikely to reduce pain (see Appendix S2). Thus, the current findings (and indeed, the priming literature more generally) offer additional evidence suggesting that placebo effects can occur via associative conditioning, unmediated by expectancies about the effect of treatment.

The current finding is qualified by several limitations. First, the use of experimentally induced pain and the characteristics of the sample (healthy, mostly female undergraduates) limit the generalizability of the finding; it is not clear whether a comparable effect would be observed in people suffering from pain in natural contexts. Second, replication of the current effect is needed to understand the reliability of the novel paradigm used here (but see [12]). Third, although experimenters were blind to experimental hypotheses, they were not blind to condition. It is unlikely, though possible, that the experimenters guessed the experimental hypothesis (see Appendix S2; see also [12]). Fourth, although the observed effect was not dependent on changes in positive or negative affect, the specific mechanism by which it occurs is unclear. For example, exposure to ibuprofen could have primed the concept of analgesia (e.g., [42], [43]) or activated the goal of avoiding or tolerating pain [44], [45]. Alternatively, exposure to ibuprofen could have primed a mental procedure by which pain could be reduced [46] or initiated a mental simulation of consuming ibuprofen [47]. All of these processes are plausible routes by which the effect could have taken place; given their potentially different theoretical and clinical implications, they are important topics for future investigation.

In sum, the current work demonstrates that objects in the environment can nonconsciously influence pain tolerance. This suggests that priming approaches could hold promise as efficient clinical interventions, and thus future work could adopt similar paradigms to explore the potential clinical utility of priming-based interventions. Moreover, the current finding strengthens the evidence that placebo effects can occur in the absence of expectancies, and suggests that priming procedures could be applied to refine understanding of the mechanisms involved in placebo responses. More generally, that external and contextual factors such as environmental cues can influence pain tolerance implies the potential for an array of new approaches to pain management.

## Supporting Information

Appendix S1 **Product Evaluation Questionnaire for key object.**
(DOC)

Appendix S2 **Additional studies.**
(DOC)
