# Supplementary material for: Handling Ibuprofen Increases Pain Tolerance and Decreases Perceived Pain Intensity in a Cold Pressor Test
Source: PLoS One. 2013 Mar 4;8(3):e56175. doi: 10.1371/journal.pone.0056175 (PMC3587636; doi:10.1371/journal.pone.0056175)
Supplement: Appendix S1 — Product Evaluation Questionnaire for key object. (DOC) [file pone.0056175.s001.doc]

Appendix A

Product Evaluation Questionnaire (key object)

Please pick up the container in front of you, and hold it in your hand. As you answer these questions, we want you to get a good impression of the product.

While holding the product answer the following questions by circling the response that best applies. Please circle only one answer:

**(1)Overall, how well-designed is the container this product comes in?**

1 2 3 4 5 6 7

(Not well-designed) (Very well-designed)

**(2) How well is the weight of the product distributed for the packaging it has?**

1 2 3 4 5 6 7

(Not very well) (Very well)

**(3) How well designed is the information about the product distributed on the container?**

1 2 3 4 5 6 7

(Not well-designed) (Very well-designed)

**(4) In general, what type of impression does this product give you?**

1 2 3 4 5 6 7

(Bad impression) (Good impression)

**(5) How much do you think this product costs? (Please fill in): __________________**
