# Supplementary material for: Handling Ibuprofen Increases Pain Tolerance and Decreases Perceived Pain Intensity in a Cold Pressor Test
Source: PLoS One. 2013 Mar 4;8(3):e56175. doi: 10.1371/journal.pone.0056175 (PMC3587636; doi:10.1371/journal.pone.0056175)
Supplement: Appendix S2 — Additional studies. (DOC) [file pone.0056175.s002.doc]

Appendix S2

Supplemental Studies

To examine if people indeed do not expect merely examining ibuprofen to confer pain tolerance, we conducted a supplemental study. Fifty-nine undergraduate volunteers completed a survey assessing their expectations about the effects of being exposed to various substances (e.g., coffee, water) without consuming them. For each substance, an item assessed whether the effect one would normally experience from consuming the substance (e.g., energy, quenched thirst) would be experienced by merely handling, seeing, or smelling the substance. The key item asked: “Taking ibuprofen makes people feel less pain. In your opinion, how plausible is it that just picking up a bottle of ibuprofen (Advil) could make someone feel less pain?” Half of the effects were framed in the converse direction (e.g., *less* energy, *more* thirst); each participant reported expectations about each substance only once, and the direction of the effect was counterbalanced. Thus, half of the participants answered the converse item for ibuprofen: “…how plausible is it that by picking up a bottle of ibuprofen (Advil), someone could actually feel more pain?” All items were answered on nine-point Likert scales anchored at “not at all plausible (1)” and “very plausible (9).” In an absolute sense, participants did not expect handling ibuprofen to reduce pain (*M* = 2.96 on the 9-point scale, *SD =* 1.93). If anything, participants’ expectations were in the other direction; handling ibuprofen was rated as more plausibly *increasing* pain, though this difference was only marginally statistically significant (*M* = 3.97, *SD =* 2.18; *t*(57) = 1.86, *p* = .07). Thus, participants did not expect that handling ibuprofen would reduce pain, implying that if the procedure reduced pain, this influence would take place nonconsciously.

A second supplemental study, identical to the first, was conducted; 44 undergraduate research assistants (*M_experience_* = 9.15 months) served as participants. As in the first study, participants did not expect handling ibuprofen to reduce pain (*M* = 4.12, *SD =* 2.39), nor did they expect handling ibuprofen to increase pain (*M* = 4.22, *SD =* 2.73); the difference between the two items did not approach statistical significance, *t*(42) = 0.14, *p* = .89. Thus, even research assistants actively working in laboratories did not expect handling ibuprofen to reduce pain, suggesting that the experimenters who conducted the sessions reported in the current manuscript were unlikely to have expectations about the directionality of the hypothesized effect.
